# Glycosaminoglycans and hyaluronic acid in chronic prostatitis/primary prostate pain syndrome: an evidence-grounded perspective

**DOI:** 10.3389/fsurg.2025.1753129

**Published:** 2026-01-20

**Authors:** Valerio Iacovelli, Carlo Brocca, Marco Carilli, Matteo Vittori, Michele Antonucci, Pierluigi Bove

**Affiliations:** 1 Minimally Invasive and Robotic Urology Unit, Tor Vergata University Hospital, Rome, Italy; 2 Department of Surgical Sciences, University of Rome Tor Vergata, Rome, Italy

**Keywords:** chronic pelvic pain (CPP), chronic prostatitis (CP), CS, HA, hyaluronic acid, glycosaminoglycans

## 1 Introduction

Chronic prostatitis/primary prostate pain syndrome (CP/PPPS) is defined by the European Association of Urology (EAU) as a clinical condition characterized by persistent or recurrent episodic prostatic pain (1). It falls under Category III of the National Institutes of Health (NIH) prostatitis classification (2). According to the International Continence Society (ICS) terminology for sexual health in men with lower urinary tract (LUT) and pelvic floor (PF) dysfunction, CP/PPPS forms part of the broader entity known as CP/CPPS (3). Clinically, CP/PPPS is characterized by pain typically reproducible on prostate palpation in the absence of proven infection or identifiable local pathology. Although historically referred to as “chronic prostatitis,” this terminology is increasingly considered inappropriate by expert consensus (1). CP/PPPS has a multifactorial etiology, encompassing acute pain mechanisms, chronic central nervous system dysfunction, neuropathic pain, and psychosocial factors including emotional, cognitive, behavioral, and sexual components (4). The syndrome often coexists with lower urinary tract symptoms (LUTS) and sexual dysfunction, significantly impairing quality of life (QoL) and presenting substantial therapeutic challenges. Conventional oral treatments, such as alpha-blockers, antibiotics, anti-inflammatories, and phytotherapeutics, frequently yield suboptimal results, highlighting the need for alternative or adjunctive approaches (1, 5).

Recent attention has focused on bioactive compounds with anti-inflammatory, antioxidant, and epithelial-protective properties. Among these, curcumin and quercetin have shown potential in modulating inflammatory pathways, while hyaluronic acid (HA) and chondroitin sulfate (CS) contribute to mucosal repair and maintenance of the uroepithelial barrier (6–9). Glycosaminoglycans (GAGs), including HA and CS, represent a critical component of the extracellular matrix and urothelial mucus layer, playing a key role in water binding, neutralization of urinary solutes, and epithelial defense mechanisms (9–12). Disruption of the GAG layer has been associated with chronic epithelial damage and neurogenic inflammation, suggesting a potential therapeutic role for GAG replenishment in CP/PPPS and other chronic pelvic pain syndromes (9, 13, 14).

The urothelium and prostatic epithelium share structural and functional similarities, including the presence of a glycosaminoglycan-rich protective layer (14). This has prompted consideration of glycosaminoglycans such as HA, CS, and pentosan polysulfate sodium (PPS) as potentially valuable adjunctive therapies. Given the established roles of these molecules in providing epithelial protection, modulating inflammatory activity, and attenuating nociceptive signaling within the bladder and other mucosal tissues, investigators have hypothesized that restoration or supplementation of the GAG layer may confer clinical benefits in patients with CP/PPPS.

This opinion article reviews the pathophysiological basis, available clinical evidence, and limitations of current research on GAG-based therapy and provides an interpretive discussion of how these findings may inform contemporary management strategies, with particular emphasis on the potential role of hyaluronic acid within this therapeutic landscape. Literature for this narrative opinion review was identified through targeted PubMed and Embase searches of English-language publications on CP/PPPS and GAGs. Priority was given to clinical, prospective, and mechanistic studies relevant to epithelial barrier function and chronic pelvic pain. No formal systematic review or meta-analysis was performed.

## 2 Pathophysiology and pathophysiological rationale for GAG supplementation

### 2.1 Epithelial barrier disruption and neurogenic inflammation

The pathophysiology of CP/PPPS is complex and multifactorial (1, 4, 5). The epithelial surfaces of the lower urinary tract possess a specialized structure—the mucus—intended to limit permeability, maintain tissue integrity, and protect underlying sensory and immune cells from irritative stimuli (14). The biologic activity of mucus that imparts this barrier function is generated by the highly anionic polysaccharide components (e.g., GAGs), which are extremely hydrophilic and trap water (9, 14). While anatomically distinct, the bladder urothelium and prostatic epithelium share key barrier-related features, including a glycosaminoglycan-rich surface layer, tight junctions, and exposure to potentially irritative urinary solutes. As proposed by Parsons (14), chronic pelvic pain conditions may reflect a common process of lower urinary dysfunctional epithelium with increased permeability and potassium-driven sensory activation. Although GAG layer disruption is well established in bladder pain syndrome and interstitial cystitis, evidence in the prostate remains limited, making this analogy primarily hypothesis-generating.

Composed of molecules such as HA and CS, the GAG layer contributes to hydrophilicity, charge repulsion, and selective permeability, protecting against noxious solutes such as potassium, urea, and ammonia. Damage or depletion of this layer has been well documented in bladder pain syndrome and interstitial cystitis, where it is associated with increased epithelial permeability, exposure of subepithelial nerve endings, mast cell activation, and propagation of neurogenic inflammatory cascades (14). Although direct evidence of GAG layer dysfunction within prostatic tissue remains limited, conceptual and mechanistic parallels suggest that similar processes may occur in CP/PPPS. As proposed by Parson, a name such as *lower urinary dysfunctional epithelium* could incorporate all of these diseases under a single pathophysiologic process (14). Chronic irritative stimuli—including alterations in prostatic secretions, local microtrauma, or chemical exposure from urine reflux—may disrupt epithelial integrity, increase permeability, and heighten afferent sensitivity. This can facilitate the release of inflammatory mediators and neuropeptides that promote nociception, sustain peripheral sensitization, and ultimately contribute to the transition from episodic discomfort to chronic pelvic pain.

The rationale for GAG supplementation in CP/PPPS derives from this biological framework. By restoring the protective barrier and reducing epithelial permeability, exogenous HA or CS may diminish exposure of sensory afferents to irritants. This could attenuate neurogenic inflammation, reduce pain signaling, and lower the propensity for central sensitization. While definitive demonstration in prostatic tissue is needed, the translational logic remains compelling and aligns with therapeutic successes observed in related pelvic pain conditions.

### 2.2 Anti-inflammatory and cytoprotective mechanisms

Beyond their barrier-restorative properties, GAGs exert biologically meaningful anti-inflammatory and cytoprotective effects. HA can modulate inflammatory pathways through interaction with CD44 and RHAMM receptors, influencing leukocyte migration, cytokine release, and tissue remodeling (15). Both HA and CS have demonstrated the capacity to attenuate activity of proinflammatory mediators such as TNF-α and IL-1β, reduce oxidative stress, and inhibit NF-*κ*B activation (16), thereby potentially counteracting the low-grade inflammatory milieu frequently observed in CP/PPPS.

PPS, a semisynthetic sulfated GAG, exhibits additional characteristics, including mast cell stabilization, antifibrotic effects, and modulation of heparanase activity. These properties may be relevant given the evidence implicating mast cell activation and fibrotic remodeling in chronic pelvic pain disorders (9).

Nutraceutical combinations incorporating GAGs with polyphenols such as curcumin and quercetin may offer further benefits by targeting multiple mechanistic pathways simultaneously. Curcumin has well-established antioxidant and anti-nociceptive activity, modulates MAPK and NF-*κ*B pathways, and reduces oxidative stress markers (6). Quercetin similarly exerts anti-inflammatory effects and may influence mast cell degranulation (7, 8). Together with HA and CS, these compounds may create a synergistic therapeutic environment aimed at restoring epithelial integrity, limiting inflammation, and dampening nociceptive signaling.

## 3 Clinical evidence

### 3.1 Glycosaminoglycan replenishment

The earliest investigation of PPS in 24 patients with chronic non-bacterial prostatitis was conducted by Wedrén et al. in 1985. Although global symptom improvements were modest, significant benefits were noted in musculoskeletal pain (*p* < 0.01), suggesting that PPS may exert effects beyond direct prostatic inflammation. The therapy was generally well tolerated, with mild gastrointestinal side effects observed in a minority of patients (17) (Table 1).

**Table 1:** Key clinical studies evaluating glycosaminoglycan-based therapies in CP/PPPS, including study design, sample size, intervention, duration, and principal outcomes.

Reference	Study design	Number of patients, gender, median age	GAG used	Regimen	Outcome parameters	Result
Wedren (17)	Prospective	24 ♂	PSS	100 mg PPS ore placebo two times daily, for 3 months	Score of signs symptoms	Not significant
	Randomized, double-blind placebo controlled	PPS 40.5 (24–46)				
		Placebo 38.5 (26–47)				
Nickel et al. (18)	Prospective	32 ♂	PPS	100 mg three times daily, for 6 months	SSI	SSI: 53.6 to 36.3*
		45.5 ± 11			SFQ	
					NIH-CPSI	SFQ: 28.1 to 17.9*
					QoL	
						NIH-CPSI: 14.5 to 9.2*
						QoL: 5.3 to 3.8*
Nickel et al. (19)	Prospective	100 ♂	PPS	300 mg PPS or placebo three times daily	CGI	CGI: PPS 1, placebo 0.6; *p* = 0.107
	Randomized, double-blind placebo controlled	PPS 40.8 (21–59)			NIH-CPSI	
		Placebo 37.5 (25–55)				Mean total NIH-CPSI: not significant
						NIH-CPSI QoL: −2.0 or 22% vs. −1.0 or 12%*
Iacovelli et al. (20)	Prospective	20 ♂	HA and CS	100 mg HA + 200 mg CS two times daily, for 2 months	SSI	SSI: 54 to 33*
		50 (46–51)			SFQ	
					NIH-CPSI	SFQ: 20 to 13*
					IPSS	
					QoL	
						NIH-CPSI: not significant
						IPSS: 13 to 11; *p* = 0.33
						QoL: 3 to 3.3; *p* = 0.15

CGI, clinical global improvement; CS, condroitin sulfate; HA, hyaluronic acid; IPSS, international prostatic symptoms score; NIH-CPSI, NIH-chronic prostatitis symptom pain index; PPS, pentosan polysulfate sodium; QoL, quality of life questionnaire; SFQ, symptom frequency questionnaire; SSI, symptom severity index.

* Statistically significant *P* < 0.05.

The clinical application of GAG replenishment in CP/PPPS has been explored with PPS and other formulations. In 2000, Nickel et al. reported a pilot study in which 32 men received PPS 100 mg thrice daily for 24 weeks. Significant improvements were observed in symptom frequency (Symptom Frequency Questionnaire, SFQ: 28.1–17.9), symptom severity (Symptom Severity Index, SSI: 53.6–36.3), and NIH-CPSI (National Institutes of Health Chronic Prostatitis Symptom Index) pain scores (14.5–9.2), alongside enhanced QoL measures. These results suggested potential efficacy, although the small sample size and moderate adverse events limited generalizability (18). A subsequent randomized controlled trial by the same authors enrolled 100 men to PPS 300 mg TID or placebo over 16 weeks. Clinical Global Improvement (CGI) scores favored PPS (37% vs. 18%, *p* = 0.04), although NIH-CPSI reductions did not reach statistical significance compared with placebo (*p* = 0.068), highlighting the challenges of placebo responsiveness in CP/PPPS studies (19).

### 3.2 Multicomponent nutraceutical approaches

Emerging evidence supports the use of oral nutraceutical combinations integrating anti-inflammatory and barrier-restorative agents. Iacovelli et al. prospectively evaluated 20 men receiving a formulation containing curcumin, quercetin, HA, and CS (Ialuril Soft Gels®) for 60 days. Significant reductions were observed in SSI (median 54–33 at 90 days, ∼40% reduction) and SFQ scores (median 20–13 at 90 days, ∼35% reduction). NIH-CPSI pain scores improved from 10–6 by day 30, with sustained benefit at 90 days. IPSS and NIH-CPSI LUTS domains showed partial improvement, while erectile function remained unchanged. The intervention was well tolerated with no reported adverse events (20).

These findings suggest that a multimodal oral approach may achieve clinically relevant symptom relief through complementary mechanisms, including reduction of inflammation (curcumin, quercetin) and restoration of epithelial barrier function (HA, CS). The data further support the concept that CP/PPPS is a multifactorial syndrome requiring integrated therapeutic strategies.

### 3.3 Insights for CP/PPPS management

The accumulated evidence indicates that GAG replenishment, alone or in combination with bioactive nutraceuticals, may represent a rational component of multimodal CP/PPPS management. Restoration of the epithelial barrier likely mitigates the chronic inflammatory cycle, while antioxidant and anti-inflammatory compounds modulate nociceptive signaling and tissue stress responses.

Despite promising results, several limitations temper the interpretation of available data. Many studies involve small sample sizes, short follow-up durations, and heterogeneous patient populations. Placebo responsiveness remains a significant challenge in CP/PPPS trials, particularly when evaluating subjective outcomes such as pain and urinary symptoms. This could reflect symptom variability, patient expectation, and the subjective assessment of pain and urinary symptoms. This complicates efficacy interpretation and highlights the need for well-designed, adequately powered randomized controlled trials using validated outcome measures. Furthermore, standardization of outcome measures, including NIH-CPSI, SSI, SFQ, and IPSS, is critical to facilitate comparability across studies.

Future research should prioritize large-scale randomized controlled trials with long-term follow-up, evaluation of combinatorial therapies, and incorporation of objective biomarkers of inflammation and epithelial integrity. Mechanistic studies examining the synergistic effects of anti-inflammatory, antioxidant, and barrier-restorative agents may further elucidate their role in modulating CP/PPPS pathophysiology.

## 4 Discussion

The integration of GAG replenishment and nutraceutical interventions represents an emerging paradigm in CP/PPPS management. Published evidence suggests that PPS, HA, and CS—particularly when combined with anti-inflammatory compounds such as curcumin and quercetin—can ameliorate pain severity, symptom frequency, and potentially improve QoL outcomes. These interventions target key pathophysiological mechanisms, including epithelial barrier dysfunction, chronic inflammation, and neuropathic pain pathways, distinguishing them from conventional monotherapies.

Although HA, CS, and PPS are all GAGs, they are not therapeutically equivalent (9). PPS is a semisynthetic, systemically active agent primarily studied orally in CP/PPPS, whereas HA mainly supports epithelial barrier function and local inflammatory modulation, and chondroitin sulfate contributes structural extracellular matrix support. Their clinical evidence bases and mechanisms differ and should be considered complementary rather than interchangeable.

Within this therapeutic landscape, the role of HA merits particular attention. HA is a naturally occurring, non-sulfated GAG that contributes substantially to epithelial barrier function across mucosal tissues. Its physicochemical properties—including hydrophilicity, viscoelasticity, and capacity to bind water molecules—allow it to create a protective surface layer that limits irritant penetration. By restoring this function when endogenous GAG layers are compromised, exogenous HA may reduce epithelial permeability and mitigate exposure of nociceptors to inflammatory or chemical stimuli. Furthermore, HA exerts direct anti-inflammatory effects that may be especially relevant in CP/PPPS. Its interactions with CD44 receptors influence leukocyte recruitment and activation, while its ability to modulate cytokine release may attenuate the inflammatory milieu within the prostate. The reduction of oxidative stress and suppression of NF-*κ*B activation observed in experimental studies suggest that HA may help counteract some of the fundamental pathways involved in pain amplification and chronicity. The use of HA in combination with chondroitin sulfate may provide synergistic benefits. CS contributes additional structural reinforcement to the extracellular matrix and may further stabilize epithelial surfaces. The combination has shown efficacy in bladder pain syndrome and interstitial cystitis, and while its direct evaluation in CP/PPPS remains limited, translational reasoning supports its potential usefulness.

In contrast to PPS—which possesses broader pharmacological properties including anticoagulant and antifibrotic effects—HA's actions are more specifically aligned with epithelial stabilization and localized modulation of inflammation. This specificity may prove advantageous in patients whose symptom profiles suggest a predominant epithelial or urothelial component. Symptoms such as urinary frequency, postvoid discomfort, and pain exacerbated by bladder filling could plausibly reflect GAG layer compromise and may therefore respond more favorably to HA-based interventions.

Although encouraging, the current literature remains limited by methodological constraints, including small sample sizes, short observation periods, and lack of blinded comparator arms in some studies. Placebo effects and patient heterogeneity further complicate the assessment of therapeutic efficacy. Nevertheless, the convergence of mechanistic rationale and preliminary clinical benefit provides a compelling case for continued investigation.

In conclusion, GAG-based therapies, alone or in combination with bioactive nutraceuticals, represent a promising avenue for CP/PPPS management. Multimodal approaches addressing inflammation, barrier integrity, and neuropathic mechanisms are likely to yield superior outcomes compared with conventional pharmacologic therapies. Well-designed, larger-scale randomized trials with long-term follow-up are warranted to define optimal therapeutic regimens and clarify their role in routine clinical practice.
